# Immunotherapy Associated Pulmonary Toxicity: Biology Behind Clinical and Radiological Features

**DOI:** 10.3390/cancers11030305

**Published:** 2019-03-05

**Authors:** Michele Porcu, Pushpamali De Silva, Cinzia Solinas, Angelo Battaglia, Marina Schena, Mario Scartozzi, Dominique Bron, Jasjit S. Suri, Karen Willard-Gallo, Dario Sangiolo, Luca Saba

**Affiliations:** 1 Department of Radiology, University Hospital of Cagliari, 09042 Monserrato (Cagliari), Italy; micheleporcu87@gmail.com (M.P.); lucasabamd@gmail.com (L.S.); 2 Molecular Immunology Unit, Institut Jules Bordet, Universitè Libre de Bruxelles (ULB), 1000 Brussels, Belgium; pushpamali.de.silva@gmail.com (P.D.S.); karen.willard-gallo@bordet.be (K.W.-G.); 3 Clinical and Experimental Hematology, Institute Jules Bordet, Universitè Libre de Bruxelles (ULB), 1000 Brussels, Belgium; Dominique.Bron@ulb.ac.be; 4 Department of Medical Oncology and Hematology, Regional Hospital of Aosta, 11100 Aosta, Italy; ABattaglia@ausl.vda.it (A.B.); MSchena@ausl.vda.it (M.S.); 5 Department of Medical Oncology, University Hospital of Cagliari, 09042 Monserrato (Cagliari), Italy; marioscartozzi@gmail.com; 6 Lung Diagnostic Division, Global Biomedical Technologies, Inc., Roseville, CA 95661, USA; jsuri@comcast.net; 7 AtheroPoint™ LLC, Roseville, CA 95661, USA; 8 Department of Oncology, University of Torino, 10043 Orbassano (Torino), Italy; dario.sangiolo@ircc.it; 9 Division of Medical Oncology, Experimental Cell Therapy, Candiolo Cancer Institute FPO-IRCCS, 10060 Candiolo (Torino), Italy

**Keywords:** PD-1/PD-L1, immune related pneumonitis, CT scan, immunotherapy, early diagnosis

## Abstract

The broader use of immune checkpoint blockade in clinical routine challenges clinicians in the diagnosis and management of side effects which are caused by inflammation generated by the activation of the immune response. Nearly all organs can be affected by immune-related toxicities. However, the most frequently reported are: fatigue, rash, pruritus, diarrhea, nausea/vomiting, arthralgia, decreased appetite and abdominal pain. Although these adverse events are usually mild, reversible and not frequent, an early diagnosis is crucial. Immune-related pulmonary toxicity was most frequently observed in trials of lung cancer and of melanoma patients treated with the combination of the anti-cytotoxic T lymphocyte antigen (CTLA)-4 and the anti-programmed cell death-1 (PD-1) antibodies. The most frequent immune-related adverse event in the lung is represented by pneumonitis due to the development of infiltrates in the interstitium and in the alveoli. Clinical symptoms and radiological patterns are the key elements to be considered for an early diagnosis, rendering the differential diagnosis crucial. Diagnosis of immune-related pneumonitis may imply the temporary or definitive suspension of immunotherapy, along with the start of immuno-suppressive treatments. The aim of this work is to summarize the biological bases, clinical and radiological findings of lung toxicity under immune checkpoint blockade, underlining the importance of multidisciplinary teams for an optimal early diagnosis of this side effect, with the aim to reach an improved patient care.

## 1. Introduction

The term cancer immunotherapy refers to a wide spectrum of therapeutic strategies exploited to harness the immune system to fight against tumors. Immunotherapy is schematically divided into passive and active strategies [1,2,3]. 

Passive immunotherapy approaches include compounds that use immunological mechanisms passively generated in the host. They are: (1) engineered monoclonal antibodies (mAbs), able to bind to specific antigens (Ags) expressed by tumor cells (for example: trastuzumab, the anti- Human Epidermal Growth Factor Receptor 2 (HER2) mAb and rituximab, the anti-cluster of differentiation (CD) 20 Ag ubiquitously expressed by B lymphocytes); (2) chimeric Ag receptor (CAR) T cells (combining the Ag-binding properties of Abs with the cytolytic and self-renewal capacity of T cells); (3) lymphokine-activated killer (LAK) cells (highly cytotoxic activated natural killer (NK) cells and cytokine induced killer T cells) and (4) tumor-infiltrating lymphocyte (TIL) therapy andthe adoptive cell transfer (ACT) (obtained by removing some of patient’s own immune-system cells, growing them in the laboratory, and infusing the cultured cells back into the patient).

Active immunotherapy strategies are able to directly activate the immune system against tumor cells. They are: (1) recombinant cytokines; (2) vaccines; (3) Ag-loaded dendritic cells (DCs), for their ability to induce potent Ag-specific T cell responses [4]; and (4) immunomodulatory engineered mAbs targeting immune checkpoint molecules, named immune checkpoint blockade (ICB) that can be inhibitory and co-stimulatory. However, controversies still exist in the classification of ICB as being an active or passive form of immunotherapy [5]. Indeed, some of these immunomodulatory mAbs prevent crucial inhibitory pathways of the immune system, whose main physiological role is to modulate the activation of the immune response. ICB acts by promoting the activation and proliferation of T-cells against tumor cells [6,7]. ICB mAbs have the ability to rescue dysfunctional T cells, compared to exhausted or inactive T cells, whose function is kept in check by negative signals. This is different from mAbs binding to specific Ags expressed by tumor cells (a form of passive immunotherapy), whose main mechanisms of action are: (1) to prevent the intracellular signaling by blocking their specific target and (2) to activate the Ab-dependent cell-mediated cytotoxicity (ADCC).

ICB is revolutionizing treatment paradigms in oncology in a number of tumors of different histotypes [8,9,10,11], giving rise to durable responses in early and advanced settings, as monotherapy or in combination with other agents, including chemotherapy [12,13,14,15,16]. Remarkably, these treatments have also been proven to improve or maintain health-related quality of life [17]. Beside the good efficacy of ICB, the use of immunotherapy in clinical practice is associated with typical adverse events (AEs) related to the hyper-activation of the immune system, leading to the appearance of autoimmune reactions. Additionally, some fatal toxic immune effects have been reported with the use of these drugs [18] highlighting the need of an early diagnosis and consequently an early management.

Adverse reactions due to ICB can be divided into: infusion reactions, immune-related AEs (irAEs) and AEs of special interests (AEoSI) according to the recent European Society for Medical Oncology (ESMO) guidelines published with the aim to guide the management of toxicities from immunotherapy [19]. Current data show the widespread use of ICB in multiple tumor types with a variety of combinations, which reflects the large and fast growing number of patients at risk for irAEs [20,21]. Once the patients show any AE it is advisable to discontinue therapy and/or to administer immunosuppressive agents (such as corticosteroids and other drugs) [22]. Thus, it is critical to gain experience with the different manifestations of irAEs in order to detect them and properly manage treated patients. 

In this work we will review the main biological bases of ICB mechanisms of action, focusing on the possible development of AEs in the lung. We will further discuss diagnostic challenges including differential diagnosis at imaging with the main radiological patterns for an early recognition.

## 2. Immune Checkpoint Blockade: Biological Bases for its Use in Cancer Immunotherapy

**Figure 1:**
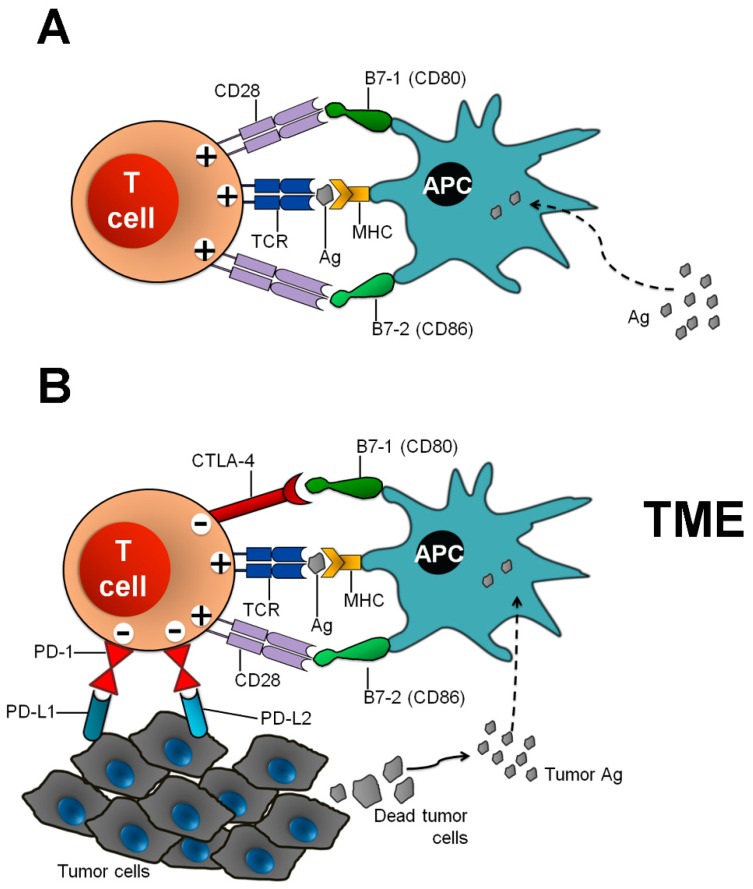
(**A**) T cell activation and CTLA-4 and PD-1 checkpoints in the regulation of antitumor T cell responses. APC presenting a processed foreign Ag on its MHC (I or II) molecule and this Ag may be recognized by the TCR on naïve T cells. To activate these naïve T cells and for effective T cell response, a secondary signal is required. This signal is provided by co-stimulatory molecule CD28 and its interaction with ligands B7-1 (CD80) and B7-2 (CD86) on professional APCs. (**B**) During strong TCR response in the tumor microenvironment due to continuous tumor Ag presentation by APCs, CTLA-4 expression is upregulated by increased transport to the cell surface from intracellular stores and decreased internalization. CTLA-4 competes with CD28 for binding of B7-1 (CD80) and B7.2 (CD86) molecules. Increased CTLA-4:B7 binding can result in a net negative signal, which limits T cell activation, proliferation, effector functions and survival. In addition, PD-1 also inhibits T cell responses after interaction with its ligands PD-L1 or PD-L2 on tumor cells (or stromal and other immune cells). CTLA-4: cytotoxic T-lymphocyte–associated antigen 4; PD-1: programmed cell death-1; PD-L1: programmed death ligand-1; PD-L2: programmed death ligand-2; MHC: major histocompatibility complex; TCR: T cell receptor; APC: antigen presenting cell, Ag: antigen, TME: tumor microenvironment.

The immune system plays a fundamental role in the host defense against foreign agents. It also warrants the avoidance of autoimmunity, which can be caused by the persistence of self-reactive T-lymphocyte clones that survived after the central thymic selection, becoming able to escape to the periphery, potentially generating inflammatory reactions against self-Ags. Noteworthy, the specific recognition by the T-cell receptor (TCR) of human leukocyte Ag (HLA)-presented Ags (first signal) by either Ag presenting cells (APCs) or by target cells is a first crucial but not sufficient step for an effective activation of T lymphocytes. A second positive signal, i.e., the binding of the co-stimulatory receptor CD28 to the ligands B7-1 (CD80) and B7-2 (CD86) on APCs, is needed for a correct priming and elicitation of Ag-specific immune-response (Figure 1).

The co-inhibitory receptor cytotoxic T-lymphocyte antigen (CTLA)-4 competes with CD28 for ligand binding or directly delivers a negative signal to T cells, preventing excessive immunity and protecting from autoimmunity [23,24,25]. The CTLA-4 mediated immune checkpoint is induced at the time of T-cell initial response to Ags, the priming phase taking place in lymph nodes. CTLA-4 is predominantly found in Foxp3^+^ regulatory T (Treg) cells or activated conventional T cells [23,26,27]. Naïve and memory T cells express high levels of CD28 but do not express CTLA-4 on their cell surface. In contrast, in these cells CTLA-4 is stored in intracellular vesicles and is transported to the cell surface only after TCR triggering by an Ag encounter [28] (Figure 1B). Harnessing immune responses against cancer by ICB was first realized using anti-CTLA-4 Abs, and has opened a new era for cancer immunotherapy [25]. Ipilimumab, a recombinant human immunoglobulin (Ig) G1 mAb and tremelimumab, a human IgG2 mAb, have both been tested in patients diagnosed with diverse advanced stage cancers [28,29,30] and are now considered for use in earlier stages of diseases, particularly in melanoma [31,32,33]. Alongside the benefits, studies demonstrated a broad variety of irAEs occurring in 60–65% of the patients. The breadth of irAEs is probably consistent with the biological role of CTLA-4 in the maintenance of polyclonal immune self-tolerance.

A number of co-signaling receptors (inhibitory and co-stimulatory) tightly regulate every step of T cell-mediated immunity, and these receptors are usually expressed on the surface of immune cells. Interactions between receptors and respective ligands generate cell-to-cell signals that control the outcome of T cells encountering with Ags [34,35]. Inhibitory receptors are able to modulate the duration and amplitude of physiological immune responses, acting for the maintenance of self-tolerance and for minimizing tissue damage caused by excessive inflammatory processes in peripheral tissues. Indeed, tissue damage is considered a physiological immune response because it can induce innate immune compartments. Among the inhibitory immune checkpoint molecules, the pathway consisting of the programmed cell death-1 (PD-1) receptor (CD279) and its ligands programmed death – ligand 1 (PD-L1; B7-H1, CD274) and PD-L2 (B7-DC, CD273) induces and maintains peripheral tolerance of T cells (Figure 1B). However, the PD-1:PD-L1/L2 pathway mediates potent inhibitory signals to hinder the proliferation and function of effector T cells, having negative effects on anti-tumor immunity [36,37]. Therapeutic targeting of this pathway with the use of mAbs that prevent these negative interactions has resulted in rescuing T-cell activity against tumors. PD-1 is found on activated CD4^+^ and CD8^+^ T cells, B cells, monocytes, NK cells and DCs [11]. Its expression can also be induced on APCs and myeloid CD11c^+^ DCs [38]. Some cytokines, i.e., interleukin-2 (IL-2), IL-7, IL-15 and IL-21, induce PD-1 expression on T cells [39]. In macrophages, interferon (IFN)-sensitive responsive element (ISRE) and STAT1/2 regulate the constitutive and IFN-α-mediated PD-1 expression [40]. PD-1 can also be selectively induced on myeloid DCs by *Listeria monocytogenes* infection or by Toll-like receptor 2 (TLR2), TLR3, TLR4, or NOD ligation, but it is inhibited by IL-4 and TLR9 [41]. PD-1 expression is also upregulated and sustained on exhausted *vs*. dysfunctional virus-specific T cells during chronic viral infections, preventing their proliferation and function in clearing the virus [42].

The major role of the PD-1 pathway is to regulate inflammatory responses in tissues by T cells recognizing Ags in the periphery (effector phase). Activated T cells up-regulate PD-1 and continue to express this receptor in tissues. In the setting of a chronic Ag exposure and a chronic stimulation from cytokines (signal 3), excessive induction of PD-1 on T cells can induce an exhausted or anergic state [42,43]. Meanwhile, inflammatory signals also induce the expression of PD-1 ligands, whose role is to down-regulate the activity of T cells and to limit collateral tissue damage. The ligands for PD-1 have distinct expression patterns. They can be expressed by immune, stromal and tumor cells (Figure 1B) [36,44,45]. PD-Ls mediate potent inhibitory signals after ligation with PD-1 expressed on T lymphocytes, causing a detrimental effect on anti-tumor immunity by allowing the tumor cells to escape from immunosurveillance. Identification of PD-Ls and confirmation of their interaction with their receptor established PD-1 as a negative regulator of immune responses.

PD-L1 is expressed on T and B cells, DCs, macrophages and bone marrow-derived mast cells in humans [45,46]. In addition, PD-L1 is expressed on a wide variety of non-hematopoietic cells including lung, vascular endothelium, fibroblastic reticular cells, liver non-parenchymal cells, mesenchymal stem cells, pancreatic islets, astrocytes, neurons and keratinocytes [46]. It has also been shown to be expressed on placental syncytiotrophoblasts with the role of inducing fetal-maternal tolerance. PD-L1 is expressed constitutively in the cornea and retinal pigmented epithelium, and its interaction with PD-1 protects the eye from activated T cells. Interestingly, in the broad spectrum of irAEs, dysimmune conjunctivitis, scleritis, episcleritis, uveitis, blepharitis, retinitis and optic neuritis have been described in patients treated with ICB [47].

PD-L2 expression is found on activated DCs (CD1a^+^ in patients with cutaneous squamous cell carcinoma), macrophages, bone marrow-derived mast cells and on more than 50% of peritoneal B1 cells. Its expression on DCs is induced by IL-4 and granulocyte monocyte-colony stimulating factor (GM-CSF). This ligand has also emerged as a natural target for cytokine production that may induce specific effector T cells to react to autologous target cells expressing PD-L2. Also tumor cells can express PD-L2, probably in association with either a helper T (Th)2 or a Th1 response, mediated by IL-4 and IL-13 as shown in esophageal cancer [48] and with IFN-γ and glycosylation in colorectal cancer (CRC) [49]. In melanoma cells, PD-L2 responds to IFN-β and IFN-γ and is regulated through both IRF1 and STAT3, which bind to PD-L2 promoter [50]. PD-L2 expression is inversely associated with a Crohn-like lymphoid reaction in CRC probably inhibiting the development of tertiary lymphoid tissues [51].

In tumors, immune checkpoint pathways have been studied as mechanisms of immune resistance, particularly because they are able to inhibit T cells specific for tumor Ags. Many of these pathways are now being blocked by Abs or modulated by recombinant forms of ligands or receptors that are used in cancer immunotherapy and are named ICB. Anti-CTLA-4, PD-1 and PD-L1 Abs achieved European Medicines Agency (EMA) and United States (US) FDA approval for the treatment of a broad spectrum of neoplastic diseases (melanoma, non-small cell lung cancer, head and neck cancer, lymphomas, microsatellite instability-high (MSI-H) solid tumors, urothelial carcinoma, renal cell carcinoma, gastric cancer, hepatocellular carcinoma and Merkel cell carcinoma), in early and advanced settings, generating durable clinical responses in tumors of different origins [21]. Table 1 summarizes the current ICB on the market for which irAEs had been documented.

## 3. Immune Related Adverse Events in Lung Due to Immune Checkpoint Blockade

The incidence of respiratory irAEs in trials with anti-PD-1 agents equaled to up to 13%, with only 2% being grade ≥3 in trials of lung cancer [52]. In studies of patients with melanoma, the incidence of these side effects was higher when using the combination of the anti-PD-1 nivolumab plus the anti-CTLA-4 ipilimumab (from 2% and 3% in monotherapy respectively to 9% in combo trials). The most frequent irAE of the respiratory tract is pneumonitis (ir-pneumonitis), the most common side effect that leads to discontinuation of immunotherapy [52]. 

From a pathological point of view, ir-pneumonitis is a non-infective inflammation of the lung. It is not a specific single entity, but is rather a spectrum of different pathological patterns characterized by the presence of infiltrates localized in the interstitium and in the alveoli, as shown in the work by Naidoo J et al. [53]. In this study three different histologic patterns of ir-pneumonitis were identified: cellular interstitial pneumonitis, organizing pneumonia and diffuse alveolar damage [50]. The median time to onset varies between agents (earlier with nivolumab, later with the anti-PD-1 pembrolizumab) [19]. Clinical manifestations are represented by: dry cough (35%), tachypnoea and dyspnea (56%), tachycardia, cyanosis and fatigue, fever (12%), chills and chest pain (7%) [54]. The chronic form is characterized by the presence of interstitial fibrosis, collagenous thickening of the alveolar septa that can occur 6-9 months after exposure.

Differential diagnosis includes: infectious pulmonary inflammations related to viruses, or to atypical germs (i.e., *Chlamydia* or *Mycoplasma*), and interstitial inflammation following the use of chemotherapy, inhaled allergens or irritants. 

Diagnostic procedures include: lung function test, blood gas analysis, thoracic computed tomography (CT) scans [52]. Imaging may help in ruling out not ir-pulmonary disease, such as bacterial pneumonia, that typically appears as asymmetrical consolidation with air bronchogram and pleural effusion [45,46]. Resistance to antibiotic treatment, absence of microrganisms in the bronchialveolar lavage and sputum can support the diagnosis of ir-pneumonitis [45,46]. The severity of irAEs is expressed in terms of grades according to the common terminology criteria for adverse events (CTCAE), recently updated to version 5.0 [55] that takes into account mainly clinical symptoms together with radiographic alterations. This scale distinguishes the AEs in five classes, from 1 to 5 according to the degree of severity. Grades 1 and 2 are reserved respectively for mild and moderate AEs, grade 3 for severe or medically significant but not immediately life-threatening AEs, grade 4 for life-threatening AEs with urgent intervention indicated, and grade 5 for death related to AEs (Table 2).

An early diagnosis is important in order to interrupt the treatment with ICB and to start immunosuppressive agents, preferably glucocorticoids (via oral or intravenous administration) and in severe cases mycophenolate mofetil [19]. No prophylaxes exists, thus an early diagnosis and a close clinical monitoring are essential to manage this side effect. Indeed, chronic pneumonitis may lead to progressive, irreversible lung disease.

## 4. Immune Related Adverse Events in Lung: Findings at Imaging

To the best of our knowledge, few radiological and pathological studies have been conducted on ir-pneumonitis. The anamnesis and clinical history are crucial in order to suspect irAEs on radiological examinations, even if one-third of the patients can be asymptomatic, having only radiologic manifestations of pneumonitis [56]. A history of ICB treatment is necessary to diagnose irAEs. Further the radiological patterns of irAEs of the lung are not specific, and can be indistinguishable from other radiological conditions.

From a pathological and radiological point of view, few studies gave important indications on the most common features of ir-pneumonitis even if a single specific pattern was not identified [54,57]. According to what can be found in the literature, this process tends to involve prominently the pulmonary interstitium, following an alveolar damage [53,58,59,60,61]. However, one case report suggested also that focal lung infiltrate could be associated with the use of PD-1 ICB [61].

The imaging technique of choice is represented by CT because of its well-known higher sensibility and specificity in the detection of abnormal pulmonary findings if compared to conventional radiology (CR) [62,63]. In addition imaging findings of ir-pneumonitis are often present and found in patients that are asymptomatic for lung disease on programmed follow-up CT examinations. Due to the fact that the radiological appearance of ir-pneumonitis is not specific and can simulate other types of interstitial lung pneumonia, researchers tried to identify the radiological features of ir-pneumonitis. They compared ir-pneumonitis with other interstitial pneumonia radiological patterns according to the American Thoracic Society/European Respiratory Society (ATS/ERS) international multidisciplinary classification of interstitial pneumonia [64].

The first authors who described in details ir-pneumonitis were Nishino et al. [65] reporting three different case reports in 2015. Later on two retrospective studies (by Naidoo et al. [53] and Nishino et al. [58]) described specific radiological patterns of this irAE.

**Figure 2:**
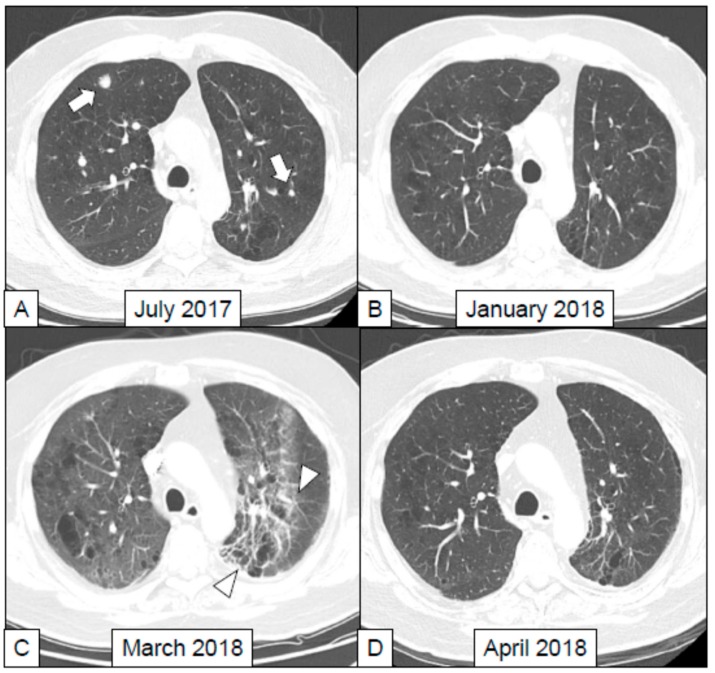
A 64 years old male, a former heavy smoker, was diagnosed with adenocarcinoma of the left lung with multiple ispilateral and controlateral lung metastases and a bone metastasis in the femur. This patient was treated with the anti-PD-1 nivolumab (3 mg/kg q2w) administered as a second line treatment for the metastatic disease. This treatment was given at the Department of Medical Oncology and Hematology, Regional Hospital of Aosta, Italy. (**A**) The lung CT scan performed in July 2017 before the beginning of immunotherapy shows the presence of pulmonary metastases in both lungs. (**B**) Those two metastases were not evident anymore in the CT scan performed 6 months later. The response was classified as partial (iPR) according to the iRECIST 1.1 criteria (42). (**C**) One month after the last CT scan, the patient developed sudden fatigue and dyspnea, with peripheral oxygen saturation equaling to 80%, with no fever and normal circulating levels of markers of systemic inflammation. A lung CT was performed in March 2018 (8 months after the beginning of immunotherapy) showing diffuse interstitial thickening associated with ground-glass pattern that was more evident in the posterior lobar regions. This aspect was suspicious for ir-pneumonitis. (**D**) The patient was treated with high dose methilprednisolone (1 mg/kg) with improvements in respiratory symptoms, and resolution of the lung pathological findings, as shown by the follow-up CT scan performed in April 2018.

In the study by Nishino et al. [58] chest CT examinations of 20 patients who suffered from ir-pneumonitis due to treatment with anti-PD-1 as single agent or in combination were retrospectively analyzed. On chest CT the extension, the distribution, the lobar involvement and the patterns of pulmonary toxicity were evaluated referring to the ATS/ERS international multidisciplinary classification of interstitial pneumonia [64]. Authors described these patterns: (1) acute interstitial pneumonia (AIP), (2) usual interstitial pneumonia (UIP), (3) cryptogenic organizing pneumonia (COP) (Figure 2), (4) non-specific interstitial pneumonia (NSIP), (5) hypersensitivity pneumonitis or (6) not applicable. In all the patient scans, ground glass opacities (GGO) were identified. In 19/20 cases GGO were associated with reticular opacities; in 12 patients with consolidations. Ir-pneumonitis showed higher extent of involvement in lower lobes, predominantly with mixed and multifocal distribution, and the COP pattern, characterized by lung opacities of variable size (from few millimeters up to several centimeters) was the most frequently observed. COP varies from GGO to lung consolidations (often accompanied by air bronchogram and mild cylindrical bronchial dilatation), with peripheral or peribronchial distribution, mainly affecting lower lobes [66]. An example of ir-pneumotis with this appearance is reported in Figure 2.

On the other hand, more recently Naidoo et al. [53] retrospectively evaluated 27 patients diagnosed with ir-pneumonitis after treatment with anti-PD-1/PD-L1 alone or in combination with anti-CTLA-4. The authors classified the radiologic features of ir-pneumonitis into five different subtypes according to the criteria for interstitial lung disease [67,68,69]: (1) COP like (Figure 2), (2) GGO, (3) interstitial, (4) hypersensitivity, and (5) pneumonitis not otherwise specified. The authors found that the GGO was the most represented pattern (10/27) but in this case they were not able to identify a dominant radiological pattern for ir-pneumonitis, since also the histological specimens obtained from 10 of these patients showed three different patterns of disease expression (see above).

It is important also to underline that ir-pulmonary toxicity can manifest with a distinct and defined pattern—different from ir-pneumonitis—the so-called “sarcoid-like pattern”. In this case hilar lymphadenopathy, associated or not with micronodules, GGO and peribronchial interstitial thickening prevalent in hilar regions, are the predominant imaging features on CT, and usually systemic symptoms are present [69,70,71,72]. 

**Figure 3:**
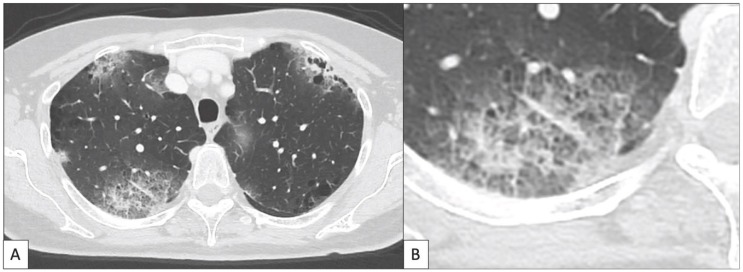
This image illustrates the main radiological features of ir-pneumonitis according to what previously published in the literature [53,54,57,58,59,60,61]. (**A**) A 70 year old woman with diagnosis of melanoma with brain metastases was treated with anti-PD-1 nivolumab (3 mg/kg q2w) administered as second line treatment for the metastatic disease. The treatment was administered at the Department of Medical Oncology of the Policlinico Universitario Duilio Casula Monserrato (CA), Italy. Seven months after the beginning of immunotherapy the patient underwent a chest CT scan for the slow, progressive appearance of fatigue and dyspnea. The chest CT showed a typical cryptogenic organizing pneumonia (COP) pattern, characterized by bilateral patchy consolidating areas with a predominantly subpleural distribution. (**B**) A detail of image A shows the area of consolidation located in the posterior segment of the upper right lobe showing ground glass opacities (GGO) and crazy paving appearance.

According to the above, we can reasonably assume that the low number of pathological and imaging studies that systemically analyzed ir-pneumonitis make difficult the identification of a single specific pattern of disease. However, the results of these studies indicate that the inflammation and the presence of infiltrates both in the alveoli and interstitium represent the leading mechanisms underlying this clinical entity, and that its main radiological expression is the presence of GGO in the context of a COP-like pattern. In Table 3 and Figure 3 we propose some elements that could help radiologist and clinicians to suspect and diagnose ir-pneumonitis.

Further studies with bigger cohorts of patients are required in order to better understand ir-pneumonitis, and a precise identification of this pattern, both with the adoption of artificial intelligence technologies such as texture analysis [73] and deep learning [74], will help radiologists to identify this condition earlier and adopt correct management. Some studies have been already done in the field of interstitial pneumonia [73,74,75,76,77] with encouraging results.

## 5. Conclusions

Ir-pneumonitis represents an unusual complication of cancer immunotherapy. Its early diagnosis represents a challenge for both clinicians and radiologists. According to the few pathological and radiological research studies found in the literature, it is reasonable thinking that ir-pneumonitis involves primarily the lung interstitium with an autoimmune process. From a radiological point of view it can manifests in different ways, but it appears similar to other types of interstitial pneumonia. The most common radiological appearance on chest CT is represented by the COP-like pattern, but the absence of a specific biomarker requires the integration of both clinical and imaging data for diagnosis. At the moment there is not a unique consensus on the optimal treatment strategy. However, this event represents the most common irAEs that leads to discontinuation of immunotherapy. Further studies will help clinicians to clarify these aspects.

## Figures and Tables

**Table 1:** Immune checkpoint blockade drugs approved in Europe and in the United States (last update: February 2019).

Immune Checkpoint Blockade	
European Medicine Agency	Food and Drug Administration
**Ipilimumab (anti-CTLA-4)**	
**Melanoma** Unresectable or metastatic disease in adults and adolescents (12 years and older) Unresectable or metastatic disease in adults in combination with nivolumab	**Melanoma** Unresectable or metastatic disease in adults and pediatric (12 years and older) patients Adjuvant treatment of patients with involvement of regional LN (>1 mm) after complete resection, including total lymphadenectomy
**RCC** 1st line treatment of adult patients with intermediate or poor risk disease in combination with nivolumab	**RCC** Intermediate or poor risk, previously untreated patients, in combination with nivolumab
	**MSI-H or dMMR CRC** Metastatic patients (adult and pediatric 12 years and older patients) that has progressed after fluoropyrimidine, oxaliplatin, and irinotecan, in combination with nivolumab
**Pembrolizumab (anti-PD-1)**	
**Melanoma** Unresectable or metastatic disease in adults Adjuvant treatment of adults with stage III disease and LN involvement who have undergone complete resection	**Melanoma** Unresectable or metastatic disease
**NSCLC** 1st line treatment of metastatic adult patients whose tumors express PD-L1 with a ≥ 50% TPS with no EGFR or ALK mutations In combination with pemetrexed and platinum chemotherapy, for the 1st line treatment of metastatic non-squamous adult patients whose tumors have no EGFR or ALK mutations Locally advanced or metastatic adult patients whose tumors express PD-L1 with a ≥ 1% TPS and who have received at least one prior chemotherapy regimen. Patients with EGFR or ALK positive mutations should also have received targeted therapy	**NSCLC** In combination with pemetrexed and platinum chemotherapy, as 1st line treatment of patients with metastatic nonsquamous disease, with no EGFR or ALK mutations In combination with carboplatin and either paclitaxel or nabpaclitaxel, as 1st line treatment of patients with metastatic squamous disease As a single agent for the 1st line treatment of patients with metastatic disease whose tumors have high PD-L1 expression (TPS ≥50%), with no EGFR or ALK mutations As a single agent for the treatment of patients with metastatic disease whose tumors express PD-L1 (TPS ≥1%) with disease progression on or after platinum-containing chemotherapy; patients with EGFR or ALK mutations should have disease progression on FDA-approved therapy for these mutations
**cHL** Adult patients with relapsed or refractory disease who have failed ASCT and BV, or who are transplant-ineligible and have failed BV	**HNSCC** Current or metastatic disease with progression on or after platinum containing chemotherapy
**UC** Locally advanced or metastatic disease in adults who have received prior platinum-containing chemotherapy Locally advanced or metastatic UC in adults not eligible for cisplatin-containing chemotherapy and whose tumors express PD-L1 with a CPS ≥ 10 Recurrent or metastatic HNSCC in adults whose tumors express PD-L1 with a ≥ 50% TPS and progressing on or after platinum-containing chemotherapy	**cHL** Adult and pediatric patients with refractory disease, or who have relapsed after 3 or more prior lines of therapy
	**PMBCL** Adult and pediatric patients with refractory disease, or who have relapsed after 2 or more prior lines of therapy
	**UC** Locally advanced or metastatic disease not eligible for cisplatin-containing chemotherapy and whose tumors express PD-L1 (CPS ≥10), or in patients who are not eligible for any platinum-containing chemotherapy regardless of PD-L1 status Locally advanced or metastatic UC who have disease progression during or following platinum-containing chemotherapy or within 12 months of neoadjuvant or adjuvant treatment with platinum containing chemotherapy
	**MSI-H cancer** Adult and pediatric patients with unresectable or metastatic MSI-H or dMMR solid tumors that have progressed following prior treatment and who have no satisfactory alternative treatment options MSI-H or dMMR CRC that has progressed following treatment with a fluoropyrimidine, oxaliplatin, and irinotecan
	**Gastric or gastroesophageal junction adenocarcinoma** Recurrent locally advanced or metastatic gastric or gastroesophageal junction adenocarcinoma whose tumors express PD-L1 (CPS ≥1), with disease progression on or after two or more prior lines of therapy including fluoropyrimidine- and platinum-containing chemotherapy and if appropriate, HER2/neu-targeted therapy
	**Cervical cancer** Recurrent or metastatic cervical cancer with disease progression on or after chemotherapy whose tumors express PD-L1 (CPS ≥1)
	**HCC** Previous treatment with sorafenib.
	**MCC** Adult and pediatric patients with recurrent locally advanced or metastatic disease
**NSCLC** 1st line treatment of metastatic NSCLC in adults whose tumors express PD-L1 with a ≥ 50% TPS with no EGFR or ALK mutations In combination with pemetrexed and platinum chemotherapy, for the 1st line treatment of metastatic non-squamous disease in adults whose tumors have no EGFR or ALK mutations Locally advanced or metastatic disease in adults whose tumors express PD-L1 with a ≥ 1% TPS and who have received at least one prior chemotherapy regimen. Patients with EGFR or ALK positive mutations should also have received targeted therapy	**NSCLC** In combination with pemetrexed and platinum chemotherapy, as 1st line treatment of patients with metastatic nonsquamous NSCLC, with no EGFR or ALK mutations In combination with carboplatin and either paclitaxel or nabpaclitaxel, as 1st line treatment of patients with metastatic squamous NSCLC As a single agent for the 1st line treatment of patients with metastatic NSCLC whose tumors have high PD-L1 expression (TPS ≥50%), with no EGFR or ALK mutations As a single agent for the treatment of patients with metastatic NSCLC whose tumors express PD-L1 (TPS ≥1%) with disease progression on or after platinum-containing chemotherapy; patients with EGFR or ALK mutations should have disease progression on FDA-approved therapy for these mutations
**cHL** Adult patients with relapsed or refractory disease who have failed ASCT and BV, or who are transplant-ineligible and have failed BV	**HNSCC** Current or metastatic disease with progression on or after platinum containing chemotherapy
**UC** Locally advanced or metastatic disease in adults who have received prior platinum-containing chemotherapy Locally advanced or metastatic disease in adults not eligible for cisplatin-containing chemotherapy and whose tumors express PD-L1 with a CPS ≥ 10	**cHL** Adult and pediatric patients with refractory disease, or who have relapsed after 3 or more prior lines of therapy
	**PMBCL** Adult and pediatric patients with refractory disease, or who have relapsed after 2 or more prior lines of therapy
	**UC** Locally advanced or metastatic patients not eligible for cisplatin-containing chemotherapy and whose tumors express PD-L1 (CPS ≥10), or not eligible for any platinum-containing chemotherapy regardless of PD-L1 status Locally advanced or metastatic disease progression during or following platinum-containing chemotherapy or within 12 months of neoadjuvant or adjuvant treatment with platinum containing chemotherapy
	**MSI-H cancer** Adult and pediatric patients with unresectable or metastatic MSI-H or dMMR solid tumors that have progressed following prior treatment and who have no satisfactory alternative treatment options MSI-H or dMMR CRC that has progressed following treatment with a fluoropyrimidine, oxaliplatin, and irinotecan
**HNSCC** Recurrent or metastatic HNSCC in adults whose tumors express PD-L1 with a ≥ 50% TPS and progressing on or after platinum-containing chemotherapy	**Gastric or gastroesophageal junction adenocarcinoma** Recurrent locally advanced or metastatic gastric or gastroesophageal junction adenocarcinoma whose tumors express PD-L1 (CPS ≥1), with disease progression on or after two or more prior lines of therapy including fluoropyrimidine- and platinum-containing chemotherapy and if appropriate, HER2/neu-targeted therapy
	**Cervical cancer** Recurrent or metastatic disease with progression on or after chemotherapy whose tumors express PD-L1 (CPS ≥1)
	**HCC** Previous treatment with sorafenib.
	**MCC** Adult and pediatric patients with recurrent locally advanced or metastatic disease
**Nivolumab (anti-PD-1)**	
**Melanoma** Advanced or metastatic disease in adults, alone or in combination with ipilimumab Adjuvant treatment of adults with involvement of LN or metastatic disease who have undergone complete resection	**Melanoma** Unresectable or metastatic disease as single agent or in combination with ipilimumab Adjuvant treatment of melanoma with LN involvement or metastatic disease who have undergone complete resection
**NSCLC** Locally advanced or metastatic disease after prior chemotherapy in adults	**NSCLC** Metastatic disease with progression on or after platinum-based chemotherapy; patients with EGFR or ALK mutations should have disease progression on FDA-approved therapy for these mutations
	**SCLC** Metastatic disease with progression after platinum-based chemotherapy and at least one other line of therapy
**RCC** After prior therapy in adult patients in combination with ipilimumab for the 1st line treatment in adults with in intermediate or poor risk advanced disease	**RCC** Advanced RCC who have received prior antiangiogenic therapy Intermediate or poor risk, previously untreated advanced RCC, in combination with ipilimumab
**cHL** Relapsed or refractory disease after ASCT and treatment with BV	**cHL** Adult patients that relapsed or progressed after HSCT and BV, or after 3 or more lines of systemic therapy that includes autologous HSCT
**SCCHN** Recurrent or metastatic disease progressing after platinum-based treatment	**SCCHN** Recurrent or metastatic disease with progression on or after a platinum-based therapy
**UC** Locally advanced unresectable or metastatic disease after failure of platinum-based treatment	**UC** Locally advanced or metastatic disease who have progression during or following platinum-containing chemotherapy, or have progression within 12 months of neoadjuvant or adjuvant treatment with platinum-containing chemotherapy
	**MSI-H or dMMR CRC** Adult and pediatric (12 years and older) patients with MSI-H or dMMR metastatic CRC progressed following treatment with a fluoropyrimidine, oxaliplatin, and irinotecan, as a single agent or in combination with ipilimumab
	**HCC** Previous treatment with sorafenib
**Atezolizumab (anti-PD-L1)**	
**NSCLC** Locally advanced or metastatic disease in adults previously treated with chemotherapy. Patients with EGFR or ALK mutations targeted treatments should also have received targeted therapy	**UC** Locally advanced or metastatic disease not eligible for cisplatin-containing chemotherapy and whose tumors express PD-L1 (PD-L1 stained tumor-infiltrating immune cells covering ≥ 5% of the tumor area), or Locally advanced or metastatic disease not eligible for any platinum—containing chemotherapy regardless of PD-L1 status, or Locally advanced or metastatic disease that have disease progression during or following any platinum-containing chemotherapy, or within 12 months of neoadjuvant or adjuvant chemotherapy
**UC** Locally advanced or metastatic disease after platinum chemotherapy Locally advanced or metastatic disease ineligible for treatment with cisplatin and whose tumours have a PD-L1 expression ≥ 5%	**NSCLC** In combination with bevacizumab, paclitaxel, and carboplatin, for the 1st line treatment, of patients with metastatic non-squamous NSCLC with no EGFR or ALK genomic tumor aberrations metastatic disease progressing during or following platinum-containing chemotherapy. Patients with EGFR or ALK genomic tumor aberrations should have disease progression on FDA-approved therapy for these aberrations
**Durvalumab (anti-PD-L1)**	
**NSCLC** Locally advanced, unresectable disease in adults, whose tumors express PD-L1 on ≥ 1% of tumor cells and whose disease has not progressed following platinum-based chemoradiation therapy	**UC** Locally advanced or metastatic disease progressng during or following platinum-containing chemotherapy Locally advanced or metastatic disease progressing within 12 months of neoadjuvant or adjuvant treatment with platinum-containing chemotherapy
	**NSCLC** Unresectable, stage III disease not progressing following concurrent platinum-based chemotherapy and radiation therapy
**Avelumab (anti-PD-L1)**	
**MCC** Adult patients with metastatic disease	**MCC** Adult and pediatric (12 years and older) patients with metastatic disease
	**UC** Locally advanced or metastatic disease progressing during or following platinum-containing chemotherapy Locally advanced or metastatic disease progressing within 12 months of neoadjuvant or adjuvant treatment with platinum-containing chemotherapy
**Cemiplimab-rwlc (anti-PD-1)**	
Not approved	**CSCC** Patients with metastatic or locally advanced disease who are not candidates for curative surgery or curative radiation

ALK: Anaplastic lymphoma kinase; ASCT: autologous stem cell transplant; BV: brentuximab vedotin; cHL: classical Hodgkin lymphoma; CPS: combined positive score; CRC: colorectal cancer; CSCC: cutaneous squamous cell carcinoma; EGFR: Epidermal growth factor receptor; FDA: food and drug administration; HCC: hepatocellular carcinoma; HNSCC: head and neck squamous cell carcinoma; LN: lymph node; Merkel Cell Carcinoma (MCC); MMR-D: mismatch repair deficient; MSI-H: microsatellite instability-high; NSCLC: non-small cell lung carcinoma; PMBCL: primary mediastinal large B-cell lymphoma; RCC: renal cell carcinoma; SCCHN: squamous cell cancer of the head and neck; SCLC: small cell lung cancer; TPS: tumor proportion score; UC: urothelial carcinoma.

**Table 2:** CTCAE grading system [55].

Common Terminology Criteria for Adverse Events (CTCAE) Grading System				
Grade		General Criteria	Criteria for Pneumonitis	Criteria for Pulmunary Fibrosis
1	Mild	Asymptomatic or mild symptoms that do not require intervention	Asymptomatic; clinical or diagnostic observations only; intervention not indicated	Radiologic pulmonary fibrosis <25% of lung volume associated with hypoxia
2	Moderate	It requires minimal, local or non invasive intervention	Symptomatic; medical intervention indicated; limiting instrumental activity of daily living (ADL)	Evidence of pulmonary hypertension; radiographic pulmonary fibrosis 25–50% associated with hypoxia
3	Severe or medically significant but not immediately life-threatening	It requires hospitalization or prolongation of hospitalization	Severe symptoms; limiting self care activity of daily living (ADL); oxygen indicated	Severe hypoxia; evidence of right-sided heart failure; radiographic pulmonary fibrosis > 50–75%
4	Life-threatening consequences	It requires urgent intervention	Life-threatening respiratory compromise; urgent intervention indicated (i.e., tracheotomy or intubation)	Life-threatening consequences (i.e., hemodynamic/pulmonary complications); intubation with ventilatory support indicated; radiographic pulmonary fibrosis >75% with severe honeycombing
5	Death	Death related to adverse event (AE)	Death	Death

**Table 3:** Suggested criteria for diagnosis of ir-pneumonitis.

Suggested Criteria for Diagnosis of ir-Pneumonitis	
Clinical criteria	History of immune checkpoint blockade (ICB) treatment
	Symptoms and/or radiological evidence of pneumonitis
	Resistance to antibiotic treatment and absence of microrganisms in the bronchoalveolar lavage and sputum
	Exclusion of other possible etiologies
Radiological criteria	Computed tomography (CT) findings of interstitial pneumonia, particularly in presence of:
	cryptogenic organizing pneumonia (COP) -like pattern
	ground glass opacities (GGO)
	“sarcoid-like” pattern
